# Clinical efficacy and safety of maintenance therapy for advanced non-small cell lung cancer: a retrospective real-world study

**DOI:** 10.1186/s12957-021-02340-0

**Published:** 2021-08-06

**Authors:** Xiangwei Xu, Ruya Li, Peizhen Zhu, Penghai Zhang, Jun Chen, Yongsheng Lin, Yinqiao Chen

**Affiliations:** 1 Department of Pharmacy, The First People’s Hospital of Yongkang, Yongkang, 321300 Zhejiang China; 2 Department of Pharmacy, People’s Hospital of Jinyun, Lishui, Zhejiang 323000 China; 3 Department of Medical Oncology, The First People’s Hospital of Yongkang, No. 599 jinshan West Road, dongcheng Street, Yongkang, 321300 Zhejiang China

**Keywords:** Advanced non-small cell lung cancer, Maintenance therapy, Chemotherapy, Targeted drug, Prognosis, Survival

## Abstract

**Background:**

The clinical efficacy and safety of maintenance therapy (MT) for patients with advanced non-small cell lung cancer (NSCLC) have not been determined in the real word. This retrospective study of real-world data analyzed these issues in patients with advanced NSCLC and stable or responsive tumors after 4–6 cycles of first-line chemotherapy.

**Methods:**

We classified 158 patients into MT (34 IIIB and 37 IV stage) and non-MT (47 IIIB and 40 IV stage) groups and then compared the clinical outcomes of progression-free survival (PFS) and overall survival (OS). The influences of maintaining chemotherapy or targeted drugs, regimens, and duration on PFS were also investigated. Prognostic factors for OS were identified by univariate and multivariate analyses.

**Results:**

Among the patients, 71 received MT and 87 did not. The median PFS and OS were significantly prolonged in the MT group than non-MT group (5.6 and 14.2 vs. 2.8 and 9.8 months, respectively; both *p* < 0.0001). The PFS was extended when patients were maintained with targeted drugs compared with chemotherapy, > 4 cycles of chemotherapy, and targeted drugs for > 3 months (all *P* < 0.0001). Patients with adenocarcinoma and without distant metastasis derived a better OS benefit from MT (*P* = 0.041 and *P* = 0.037, respectively). Multivariate analysis revealed that female sex and MT were independent prognostic factors for extended OS (*P* = 0.039 and *P* < 0.0001, respectively). The major adverse events of MT comprised tolerable hematological toxicity and gastrointestinal reactions.

**Conclusions:**

MT was advantageous and tolerable for patients with advanced NSCLC, especially those with adenocarcinomas without distant metastasis who were treated with targeted drugs, which was an independent prognostic factor for OS.

## Highlights

1. Maintenance therapy extended PFS and OS of patients with NSCLC.
2. MT with targeted drugs significantly improved PFS compared with chemotherapy.
3. Longer duration of MT with chemotherapy or targeted drugs prolonged PFS.
4. Patients with adenocarcinomas and NSCLC without distant metastasis gained more OS benefits from MT.
5. Female sex and MT were independent prognostic factors for extended OS.
6. Patients with NSCLC tolerated MT without serious adverse events.

## Background

Lung cancer remains the leading global cause of cancer-related deaths [1, 2], accounting for ~ 1.6 million annually [3]. Non-small cell lung cancer (NSCLC) accounts for 85% of all lung cancers and it histologically comprises adenocarcinoma, squamous cell carcinoma, large cell carcinoma, and other subtypes [4]. Despite significant recent progress, NSCLC is often diagnosed at advanced stages when treatment options are limited. The dawn of immune checkpoint inhibitors has notably improved the therapeutic landscape of NSCLC in terms of prolonging the life spans of patients, but real-life data remain scarce [5].

Four to six cycles of platinum-based chemotherapy are the standard first-line therapy. Patients with pStage IB-IIIA NSCLC can tolerate effective adjuvant chemotherapy with carboplatin and emcitabine well [6]. However, the prognosis for patients with advanced NSCLC is disappointing, as the 5-year overall survival (OS) rate is < 5% [7]. A meta-analysis has shown that recombinant human endostatin together with chemotherapy is safe and effective for treating advanced squamous cell lung cancer [8]. Although adding preoperative radiotherapy to chemotherapy increases the pathological response and mediastinal downstaging, it does not improve the long-term survival of patients with resectable stage IIIA/N2 NSCLC [9].

Novel therapeutic methods are urgently needed to improve the progression-free survival (PFS) and OS of these patients. At least two clinical trials have found that maintenance therapy (MT) confers survival benefits on patients with NSCLC [10, 11]. Switch and continuous MT strategies are defined as continuing drugs administered during first-line therapy, or adding drugs that differ from first-line therapy [12]. Evidence-based medicine has shown that MT with one agent can prolong the interval before advanced NSCLC progresses and results in death [13–15]. For example, continuous pemetrexed has been suggested for patients with adenocarcinomas of NSCLC, and gemcitabine is recommended for patients with squamous NSCLC [16]. Doublet MT with various treatment regimens has also been suggested, but consensus has not yet been reached [17]. Indeed, several types of MT might not be appropriate for all patients with NSCLC, such as those with inadequate organ function to tolerate the extra toxicity of MT, and some adverse events might accelerate tumor progression [18, 19]. Economic factors are also an important reason for the refusal of patients to undergo MT, and these are usually insufficiently considered in controlled clinical trials [20]. Therefore, elucidating the real-world reliability and necessity of MT for patients with advanced NSCLC is clinically meaningful.

Considering medical insurance payments and concomitant therapies, we retrospectively evaluated the clinical effects and adverse reactions of MT in patients with stage IIIB/IV NSCLC at our hospital to provide a more comprehensive understanding of the value of MT for patients with advanced NSCLC. We also compared clinical outcomes between switching with targeted drugs and continuous chemotherapy with different regimens and durations in a subgroup analysis. We then identified a subgroup of patients who might derive more benefits from MT, based on a confirmed pathological type of NSCLC and smoking history.

## Materials and methods

### Patient enrollment

This retrospective study collected clinical data from consecutive patients with stable or responding stage IIIB/IV NSCLC who received 4–6 cycles of first-line platinum-based chemotherapy between January 2013 and June 2019 at Yongkang First People’s Hospital. The inclusion criteria were as follows: age 18–80 years; histologically or cytologically confirmed stage IIIB/IV NSCLC according to the TNM classification of the Union for International Cancer Control (UICC) (8th edition) [21]; complete response (CR), partial response (PR), or stable disease (SD) after 4–6 cycles of first-line platinum-based chemotherapy, according to Evaluation Criteria in Solid Tumors (RECIST Version 1.1) [22, 23]; with or without MT after first-line chemotherapy; no other second- or third-line treatments after disease progression; no radiotherapy, immunotherapy, or other therapies before MT; no serious cardiac disease or other concomitant; and complete clinical data. Exclusion criteria comprised failure to complete 4–6 cycles of chemotherapy, progressive disease (PD) after chemotherapy, other treatments after disease progression, severe cardiac diseases, liver or kidney dysfunction, or infectious diseases and undergoing concurrent radiotherapy. All enrolled patients provided written, informed consent to participate in this study, which was approved by the Clinical Medical Ethics Committee of Yongkang First People’s Hospital (Approval code: ykyy2018-04).

### Study design

Clinical data included sex, age, marital status, smoking status, type of medical insurance, pathological type, clinical stage, metastatic sites, distal metastasis, performance status (PS) score, first-line chemotherapy drugs, antiangiogenic drugs, therapeutic effects evaluated after first-line chemotherapy, MT regimen (targeted and anti-angiogenic drugs, chemotherapy with one drug), complications during chemotherapy, and follow-up information. Patients with stable or responsive stage IIIB/IV NSCLC were also classified into the MT and non-MT groups according to whether they received MT or not after 4–6 cycles of first-line chemotherapy.

By reviewing the hospital clinical records, as well as messages and telephone follow-up, tumor response and complications were analyzed until the end of December 31, 2019. The endpoints comprised the PFS calculated from the first dose until disease progression, and OS calculated from the initial diagnosis to death or the end of follow-up, which are expressed on a monthly basis.

### Efficacy assessment

According to RECIST1.1, tumor responses compared with baseline were evaluated as CR (complete disappearance of all target and non-target lesions, with no development of new disease), PR (≥ 30% decrease in the sum of the diameters of target lesions), PD (≥ 20% increase in the sum of target lesions with an absolute increase of > 5 mm, or the appearance of at least one new lesion), and SD (shrinkage insufficient to qualify as PR or increase insufficient to qualify as PD).

### Safety assessment

All complications that developed in this study were evaluated using Common Terminology Criteria Adverse Events Version 5.0 (https://ctep.cancer.gov/protocoldevelopment/electronic_applications/docs/ctcae_v5_quick_reference_5x7.pdf), and classified as grades 1–5. The major adverse events included leukopenia, neutropenia, thrombocytopenia, anemia, nausea, vomiting, diarrhea, neurotoxicity, transaminase elevation, renal toxicity, weakness, rash, myalgia, and arrhythmia.

### Statistical analysis

Data were analyzed and figures were drafted using Excel (Microsoft Corp., Redmond, WA, USA), SPSS 17.0, (SPSS Inc., Chicago, IL, USA), and GraphPad Prism 5.0 (GraphPad Software, San Diego, CA, USA). Descriptive statistics were used for qualitative data and compared using two-sided chi-square tests. Quantitative data are expressed as means ± standard deviation and were compared using two-sided independent t-tests. Survival duration was calculated using Kaplan–Meier curves. Hazard ratio (HR) and 95% confidence interval (CI) were determined by univariate analyses with log-rank tests. Odds ratio (OR) and 95%CI were calculated using multivariate analyses of Cox regression models. Statistical significance was set at p < 0.05.

## Results

### Demographics of patients

According to the inclusion criteria, 436 patients with advanced NSCLC undergoing 4–6 cycles of chemotherapy were initially investigated, then 278 were excluded (PD after first-line chemotherapy, *n* = 152; concurrent radiotherapy, *n* = 95 and severe complications after chemotherapy, including bone marrow suppression, hepatic and renal insufficiency, *n* = 31). We finally analyzed data from 158 patients, among whom 71 (34 IIIB and 37 IV stage) underwent MT and 87 (47 IIIB and 40 IV stage) did not.

Table 1 shows that the two groups did not significantly differ in terms of sex, age, marital status, smoking status, type of medical insurance, pathological type, clinical stage, metastatic sites, number of metastatic sites, PS score, first-line chemotherapy drugs, antiangiogenic drugs, or therapeutic efficacy after first-line chemotherapy (P > 0.05). Tumor responses evaluated by RECIST v1.1 were similar after first-line chemotherapy, which resulted in 8.1% CR, 33.3% PR, and 58.6% SD in the MT group and 7.0% CR, 33.8% PR, and 59.2% SD in the non-MT group, respectively.

**Table 1:** Demographic comparison of patients in non-MT group and MT group

Index	Non-MT group (*n* = 87)	MT group (*n* = 71)	*t*/*χ* ^2^	*P* value
Age (Year, $\overline{x}$ ± s)	60.28 ± 7.78	61.63 ± 9.30	0.869	0.386
Gender (*N*)			0.079	0.778
Man	52 (59.8%)	44 (62.0%)		
Woman	35 (40.2%)	27 (38.0%)		
Marital status (*N*)			1.348	0.246
Married	76 (87.4%)	66 (93.0%)		
Non-married	11 (12.6%)	5 (7.0%)		
Smoking history			0.799	0.372
Yes	58 (66.7%)	52 (73.2%)		
No	29 (33.3%)	19 (26.8%)		
Medical insurance			2.069	0.558
New rural cooperative	37 (42.5%)	26 (36.6%)		
Residents	25 (28.7%)	21 (29.6%)		
Employees	16 (18.4%)	19 (26.8%)		
Self-pay	9 (10.4%)	5 (7.0%)		
Pathological type (*N*)			0.842	0.175
Adenocarcinomas	65 (74.7%)	46 (64.8%)		
Squamous	22 (25.3%)	25 (35.2%)		
Clinical TNM stage (*N*)			0.589	0.443
IIIB	47 (54.0%)	34 (47.9%)		
IV	40 (46.0%)	37 (52.1%)		
Metastasis site (*N*)			0.955	0.917
Contralateral lung	31 (35.6%)	27 (38.0%)		
Liver	14 (16.1%)	9 (12.7%)		
Bone	18 (20.7%)	13 (18.3%)		
Brain	17 (19.5%)	13 (18.3%)		
Others	8 (9.2%)	9 (12.7%)		
Distal metastasis (*N*)			1.313	0.252
1 ~ 2	55 (63.2%)	48 (67.6%)		
≥ 3	32 (36.8%)	23 (32.4%)		
PS score (*N*)			0.782	0.376
0 ~ 1	62 (71.3%)	55 (77.5%)		
2	25 (28.7%)	16 (22.5%)		
First-line chemotherapy drugs (*N*)			2.155	0.707
Vinorelbine + cisplatin/carboplatin	7	6		
Taxol + cisplatin/carboplatin	9	6		
Gemcitabine + cisplatin/carboplatin	26	15		
Docetaxel + cisplatin/carboplatin	20	18		
Pemetrexed + cisplatin/carboplatin	25	26		
Antiangiogenic drugs (*N*)			0.574	0.750
Recombinant human vascular endostatin	8	6		
Bevacizumab	11	12		
No	68	53		
Therapeutic efficacy			0.056	0.972
CR	7 (8.1%)	5 (7.0%)		
PR	29 (33.3%)	24 (33.8%)		
SD	51 (58.6%)	42 (59.2%)		

*MT*, maintenance treatment; *TNM*, tumor, node, metastasis; *PS*, performance statue; *CR*, complete response; *PR*, partial response; *SD*, stable disease. P value of age was calculated by two-sided *t*-test, and the rest indexes were calculated by two-sided chi-square test

### Maintenance therapy prolonged PFS

**Fig. 1:**
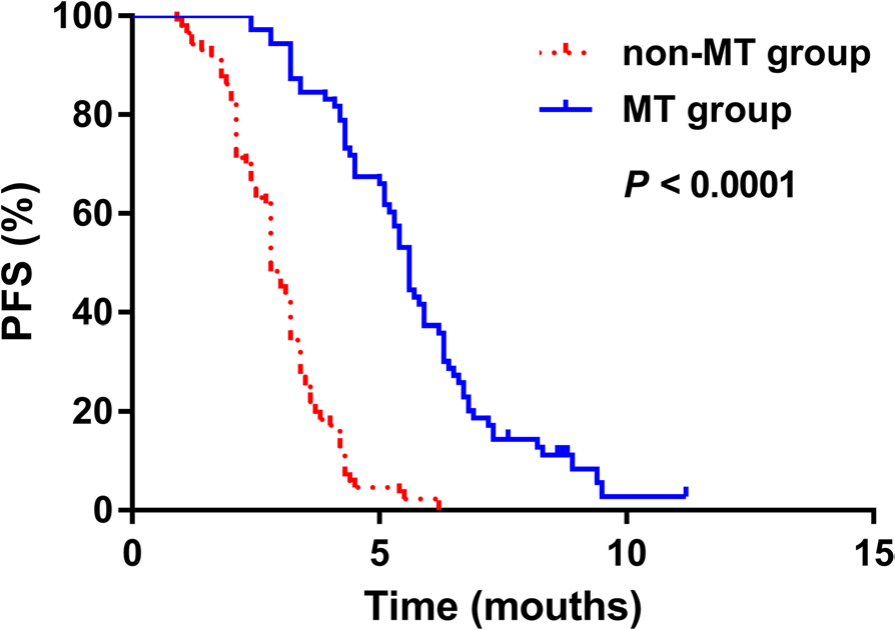
Comparison of PFS between patients with and without MT. Maintenance therapy extended PFS (*P* < 0.0001, log-rank test). MT, maintenance therapy; PFS, progression-free survival

The median PFS was 5.6 and 2.8 months in the MT and non-MT groups, respectively (Fig. 1). Therefore, MT prolonged PFS compared with clinical observation alone (HR, 7.657; 95%CI, 5.083, 11.54; *χ* ^2^, 94.81, *P* < 0.0001).

**Fig. 2:**
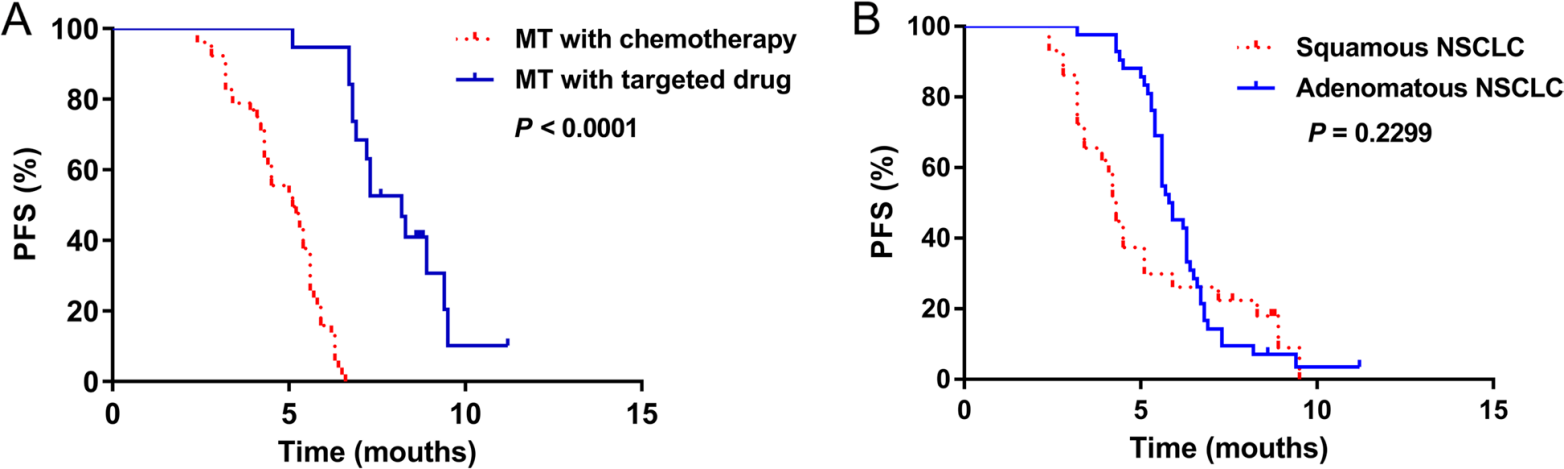
Progression-free survival. **A** Patients were maintained with chemotherapy or targeted drugs. Latter elicited more PFS improvement (*P* < 0.0001, log-rank tests). **B** Patients with adenocarcinomas or squamous NSCLC did not significantly differ (*P* = 0.230, log-rank tests). MT, maintenance treatment; NSCLC, non-small cell lung cancer; PFS, progression-free survival

Among the 71 patients who received MT, the median PFS was 5.1 and 8.2 months for 52 and 19 patients who respectively underwent MT with monotherapy and targeted drugs. The latter was significantly more effective in terms of PFS (HR, 8.327; 95%CI, 4.580, 15.14; *χ* ^2^, 48.30; *P* < 0.0001; Fig. 2A). The median PFS of 42 and 29 patients with adenocarcinoma and squamous NSCLC, respectively, was 5.85 and 4.3 months, which was not significantly different (HR, 1.399; 95%CI, 0.8087, 2.419; *χ* ^2^, 1.440; *P* = 0.230; Fig. 2B).

**Fig. 3:**
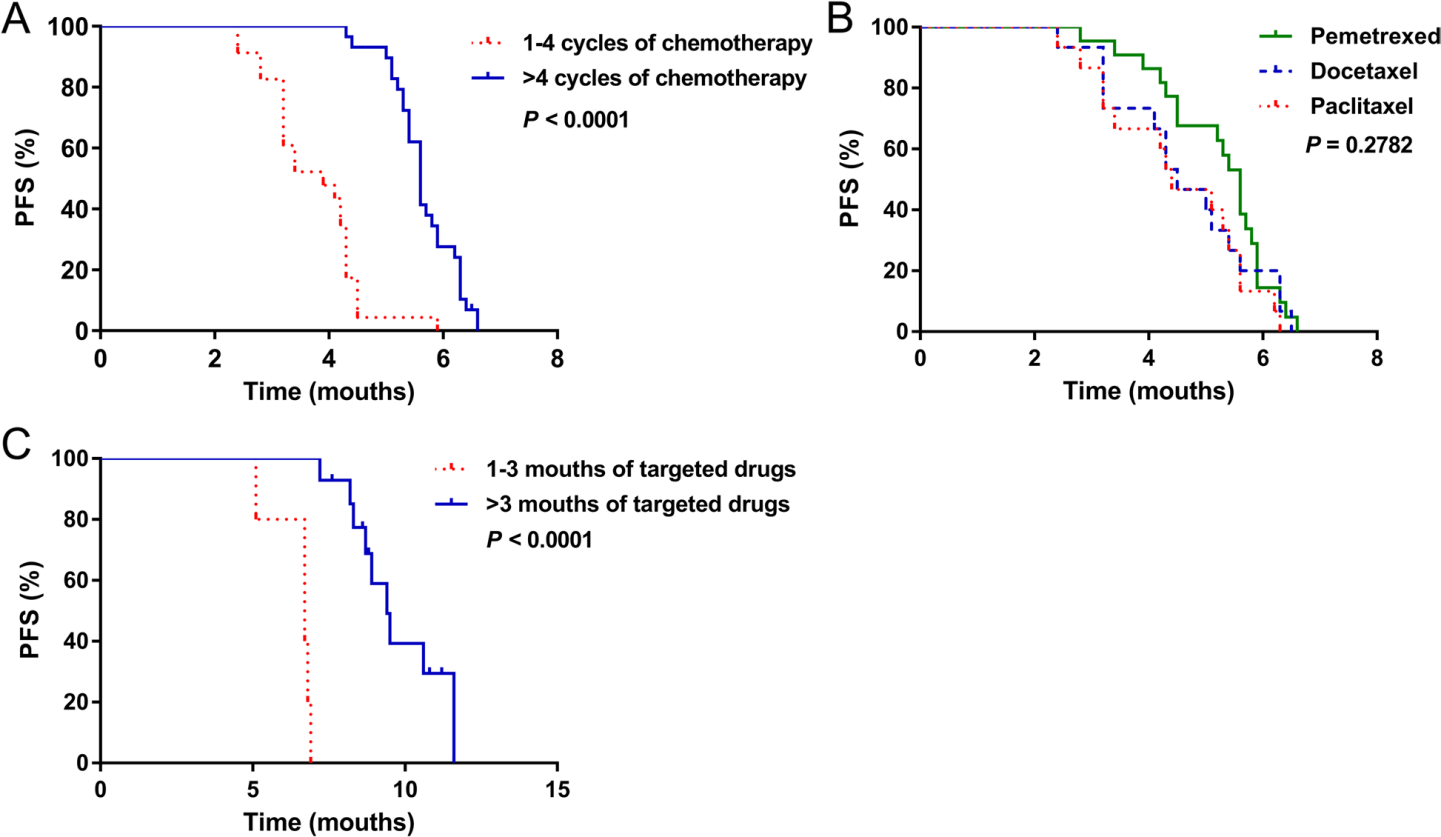
Influence of MT regimens and duration of chemotherapy or targeted drugs on PFS. Progression-free survival was prolonged after **A** > 4 cycles of chemotherapy and **C** > 3 months of targeted drugs (both *P* < 0.0001, log-rank tests). Effects of chemotherapy with pemetrexed, docetaxel, or paclitaxel on PFS did not significantly differ (*P* = 0.278, log-rank tests). MT, maintenance treatment; PFS, progression-free survival

Of the 49 patients who were maintained with chemotherapy, the median PFS of 18 patients who completed > 4 cycles was 5.6 months, which was significantly longer than that of 31 patients who completed < 4 cycles (3.9 months) (HR, 16.18; 95%CI, 6.295, 37.79; *χ* ^2^, 41.35; *P* < 0.0001; Fig. 3A). The median PFS of 22, 15, and 15 patients who received MT with pemetrexed, docetaxel, and gemcitabine was 5.6, 4.5, and 4.4 months, respectively (Fig. 3B). However, these results did not reach statistical significance (*χ* ^2^, 2.559; *P* = 0.2782).

Among the 19 patients maintaining with targeted drugs, 13, five, and one received ectinib, erlotinib, and gefitinib, respectively. The median PFS was 6.7 and 9.4 months for five and 14 patients who tolerated treatment for 1–3 and ≥ 3 months, respectively, indicating that a longer duration of targeted drug therapy resulted in a better tumor response (HR, 378.3; 95%CI, 35.79, 3,999; χ^2^ = 24.34, P < 0.0001; Fig. 3C).

### Maintenance therapy significantly extended OS

**Fig. 4:**
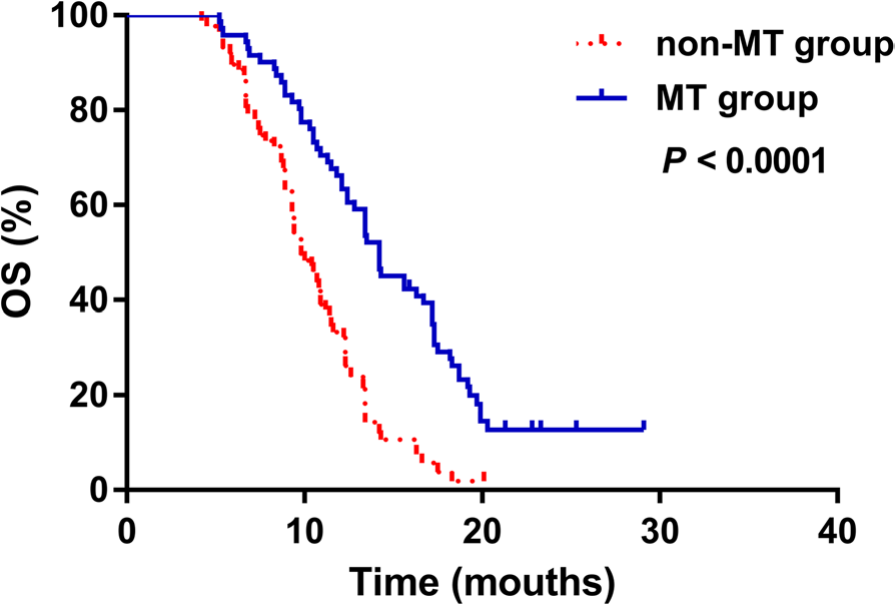
Comparison of OS between patients with and without MT. Maintenance therapy extended OS (*P* < 0.0001, log-rank tests). MT, maintenance therapy; OS, overall survival

As of December 31, 2019, 11 and five patients in the MT and non-MT groups remained alive. The OS was significantly longer for 60 eligible patients in the MT group than 82 eligible patients in the non-MT group (14.2 vs. 9.8 months; HR = 2.856, 95%CI: [1.984, 4.112]; *χ* ^2^ = 26.38, *P* < 0.0001; Fig. 4).

**Fig. 5:**
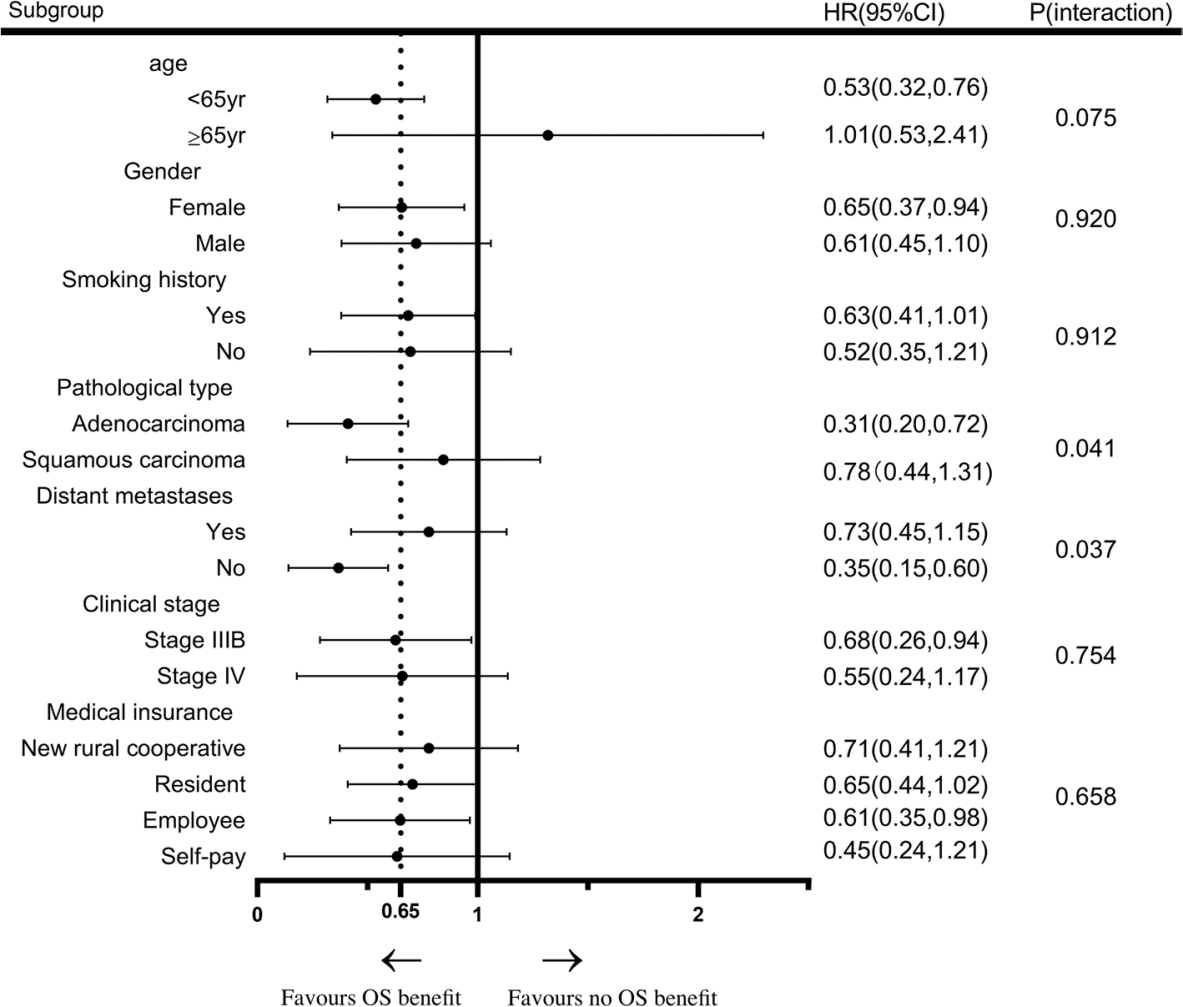
Univariate analysis of OS in patients with MT. Age, sex, smoking history, pathological type, distant metastasis, clinical stage, and medical insurance were analyzed. Patients with adenocarcinoma and without distant metastasis gained more OS benefit from MT (*P* = 0.041 and *P* = 0.037, respectively; log-rank tests). MT, maintenance therapy; OS, overall survival

Univariate analysis of OS in the MT group showed that pathological type and distant metastasis significantly influenced the clinical outcome of MT (*P* = 0.041 and 0.037, respectively). Thus, patients with adenocarcinoma (HR, 0.31; 95%CI, 0.20, 0.72) and without distant metastasis (HR, 0.35; 95%CI, 0.15, 0.60] were more likely to benefit from MT. However, OS did not significantly differ with respect to age (age ≥ 65 vs. < 65 years: *p* = 0.075), sex (*p* = 0.920), smoking history (never vs. formerly: *p* = 0.912), clinical stage (stage IIIB vs. IV: *p* = 0.754), and type of medical insurance (*p* = 0.658; Fig. 5).

Multivariate analysis of all eligible patients using Cox regression models showed that sex and MT were independent factors affecting the OS of patients with NSCLC (Table 2). Briefly, the prognosis was worse for men than women, and they had a 1.335-fold higher risk of death than women (*P* = 0.039). MT was a favorable prognostic factor for risk of death, which was 0.412-fold higher than that without MT (*P* < 0.0001).

**Table 2:** Multivariate analysis through Cox regression model for OS in advanced NSCLC patients

Variate	Classification	Regression coefficient	Standard error	*P* value	Odds ratio	95% CI
Maintenance	Yes/no	− 0.854	0.238	< 0.0001	0.412	0.22–0.59
Gender	Woman/man	0.678	0.678	0.039	1.335	1.08–3.84
Clinical stage	IIIb/IV	0.732	0.732	0.065	1.886	0.97–3.06
Age	< 65 / ≥ 65 years	0.398	0.398	0.078	1.248	0.78–1.85
Distant metastasis	No/yes	0.232	0.232	0.148	1.325	0.44–2.12
Pathological type	Adenocarcinomas/squamous	0.328	0.328	0.237	1.433	0.81–1.97
Smoking history	No/yes	− 0.258	− 0.258	0.468	0.680	0.54–2.38

*P* value was calculated by Cox regression model and odds ratio was showed as the ratio of risk of death between the two classifications in each variate

### Adverse events of MT

The major adverse events of MT were hematological toxicity of leukopenia, neutropenia, thrombocytopenia, and anemia and digestive tract reactions of nausea, vomiting, anorexia, diarrhea, neurotoxicity, renal toxicity, weakness, myalgia, arrhythmia, elevated transaminase, and rash. Table 3 shows the rates of mild to moderate (grades 1 and 2) or severe (grades 3 and 4) adverse events. No serious adverse reactions occurred, and the status of all patients returned to normal after symptomatic treatment.

**Table 3:** Incidence rate and severity of adverse events in maintenance therapy

Adverse events	Grade	Cases (incidence rate %)
Leukopenia	Grade 1–2	27 (38.03%)
	Grade 3–4	19 (26.76%)
Neutropenia	Grade 1–2	23 (32.39%)
	Grade 3–4	12 (16.90%)
Thrombocytopenia	Grade 1–2	17 (23.94%)
	Grade 3–4	7 (9.86%)
Anemia	Grade 1–2	31 (43.66%)
	Grade 3–4	12 (16.90%)
Nausea	Grade 1–2	20 (28.17%)
	Grade 3–4	3 (4.23%)
Vomiting	Grade 1–2	18 (25.35%)
	Grade 3–4	2 (2.82%)
Diarrhea	Grade 1–2	7 (9.86%)
	Grade 3–4	2 (2.82%)
Neurotoxicity	Grade 1–2	4 (5.63%)
	Grade 3–4	1 (1.41%)
Transaminase elevation	Grade 1–2	13 (18.31%)
	Grade 3–4	2 (2.82%)
Renal toxicity	Grade 1–2	9 (12.67%)
	Grade 3–4	3 (4.23%)
Weakness	Grade 1–2	33 (46.47%)
	Grade 3–4	5 (7.04%)
Rash	Grade 1–2	9 (12.67%)
	Grade 3–4	3 (4.23%)
Myalgia	Grade 1–2	3 (4.23%)
Arrhythmia	Grade 1–2	2 (2.82%)

## Discussion

Patients with stable or responsive NSCLC after 4–6 cycles of first-line chemotherapy do not need treatment before tumor progression [24, 25]. However, NSCLC can progress in such patients during a short period of rest, and rapidly deteriorating disease can negatively affect the likelihood of receiving second-line therapy. Large clinical trials of advanced NSCLC, including ECOG4599 [7], FLEX [26], and others have found that only 50% of patients given first-line therapy undergo second-line therapy. Thus, MT aimed at controlling NSCLC to prolong PFS has recently attracted attention [27]. Although MT is an innovative strategy for treating NSCLC, it should not be considered as routine therapy for patients who are intolerant or insensitive to previous or additional agents [28]. Some patients decline MT due to economic factors [20]. Consequently, a subset of patients is unable to receive MT (non-MT group). To better understand the application of MT to patients with advanced NSCLC in the real world, we compared the clinical outcomes of PFS and OS between patients with and without MT, to provide a more reliable basis for clinical MT of NSCLC.

This retrospective study found that MT led to better PFS and OS in the real world situation. Furthermore, univariate and multivariate analyses suggested that prolonged MT with targeted drugs, adenocarcinoma, and no distant metastasis were potentially favorable characteristics of patients who were likely to benefit from or be appropriate for MT. Female sex and MT were independent prognostic factors for better OS. Collectively, these data provide a reference value for clinical MT of advanced NSCLC.

MT as a potential effective measure to inhibit tumor progression has improved PFS or OS [11, 29]. However, various treatment strategies such as targeted drugs, chemotherapy, immunotherapy, and anti-angiogenic agents might influence different outcomes, [30–32]. Most of our patients who received initial chemotherapy had no driving gene mutations; however, patients who developed gene mutations later might turn to MT with targeted drugs. Classical randomized controlled trials of tyrosine kinase inhibitors (TKIs) against epidermal growth factor receptor (EGFR), including SATURN, INFORM, and EORTC008021, as well as a retrospective study, have confirmed that EGFR-TKI is more effective than a placebo [33–36]. The National Comprehensive Cancer Network (NCCN), Cancer Care Ontario (CCO) and Chinese Society Clinical Oncology (CSCO) have proposed advanced biomarker detection for patients with unresectable NSCLC [16, 37, 38]. A recent study found that patients with NSCLC adenocarcinomas gained more benefits on PFS and OS from MT because of the high incidence of driving gene mutations and the access to effective targeted drugs [12]. However, the present study did not find a statistical difference in PFS between adenocarcinomas and squamous NSCLC. This might have been due to the small sample size.

Drug resistance can develop with increasing cycles of chemotherapy [39]. We found that > 4 cycles of MT with chemotherapy were more effective than fewer cycles, which might differ from initial chemotherapy. This could be explained by the fact that regular and long-term MT with cytotoxic drugs can slowly but persistently control tumor proliferation to provide better tumor responses to treatment, better patient tolerance, and a better quality of life [40]. Pemetrexed, gemcitabine, and docetaxel improve PFS, and pemetrexed can also ameliorate OS [29, 41]; we found little distinction, which again, might have been due to the small sample size.

Although the adverse events of MT were tolerable in the present, as described in previous studies [42, 43], the MT strategy should comprehensively consider the status of the patients, drug resistance, and the prognostic profit in a rational subpopulation [19, 44, 45]. For example, patients with advanced NSCLC often develop distant metastases in the brain accompanied by neurological symptoms, in bone with osteodynia, or the liver with hepatic dysfunction [46]. This could lead to poor compliance and tolerability, thus negating the efficacy of MT. This corresponded with the results of our univariate analysis of OS, which showed that distant metastasis would limit the benefit for patients with NSCLC. Our Cox regression model to predict potential targeted subpopulations of NSCLC identified the independent predictors of female sex and MT, which also reliably confirmed the value of MT for NSCLC.

However, this study retrospectively assessed the clinical efficacy mainly based on imaging data and inpatient medical records, which lacked the double-blind and rigorous nature of a prospective investigation. The small subpopulations restricted the power of comparison, which will need enlarging to ensure the selection of appropriate patients for statistical analysis.

In conclusion, this clinically meaningful retrospective analysis of MT efficacy after first-line chemotherapy for advanced NSCLC provides real-world evidence that supports the survival benefit of MT.

## Data Availability

Not applicable.

## Abbreviations

CCO: Cancer Care Ontario
CR: Complete response
CSCO: Chinese Society Clinical Oncology
EGFR: Epidermal growth factor receptor
MT: Maintenance therapy
NSCLC: Non-small cell lung carcinoma
NCCN: National Comprehensive Cancer Network
OS: Overall survival
PD: Progressive disease
PFS: Progression-free survival
PR: Partial response
RECIST: Evaluation Criteria in Solid Tumors
SD: Stable disease
TKI: Tyrosine kinase inhibitors
UICC: Union for International Cancer Control
